# Amplifier spurious input current components in electrode-electrolyte interface impedance measurements

**DOI:** 10.1186/1475-925X-4-22

**Published:** 2005-03-29

**Authors:** Carmelo J Felice, Rossana E Madrid, Max E Valentinuzzi

**Affiliations:** 1 Departamento de Bioingeniería (DBI) Facultad de Ciencias Exactas y Tecnología (FACET) Universidad Nacional de Tucumán (UNT), Argentina; 2 Instituto Superior de Investigaciones Biológicas (INSIBIO) Consejo Nacional de Investigaciones Científicas y Técnicas (CONICET) Tucumán City, Argentina

**Keywords:** AISI 304, amplifier drift, impedance microbiology, operational amplifier, reactive component, resistive component

## Abstract

**Background:**

In Impedance Microbiology, the time during which the measuring equipment is connected to the bipolar cells is rather long, usually between 6 to 24 hrs for microorganisms with duplication times in the order of less than one hour and concentrations ranging from 10^1 ^to 10^7 ^[CFU/ml]. Under these conditions, the electrode-electrolyte interface impedance may show a slow drift of about 2%/hr. By and large, growth curves superimposed on such drift do not stabilize, are less reproducible, and keep on distorting all over the measurement of the temporal reactive or resistive records due to interface changes, in turn originated in bacterial activity. This problem has been found when growth curves were obtained by means of impedance analyzers or with impedance bridges using different types of operational amplifiers.

**Methods:**

Suspecting that the input circuitry was the culprit of the deleterious effect, we used for that matter (a) ultra-low bias current amplifiers, (b) isolating relays for the selection of cells, and (c) a shorter connection time, so that the relays were maintained opened after the readings, to bring down such spurious drift to a negligible value. Bacterial growth curves were obtained in order to test their quality.

**Results:**

It was demonstrated that the drift decreases ten fold when the circuit remained connected to the cell for a short time between measurements, so that the distortion became truly negligible. Improvement due to better-input amplifiers was not as good as by reducing the connection time. Moreover, temperature effects were insignificant with a regulation of ± 0.2 [°C]. Frequency did not influence either.

**Conclusion:**

The drift originated either at the dc input bias offset current (I_os_) of the integrated circuits, or in discrete transistors connected directly to the electrodes immersed in the cells, depending on the particular circuit arrangement. Reduction of the connection time was the best countermeasure.

## Background

Measurements carried out with bipolar electrodes can be modeled by a series circuit composed of two impedances, one representing the electrolytic medium (Z_m_) and another taking into account the interface (Z_i_) between the former and the metal itself. From a physical point of view, the interface region extends from the larger rugosities of the electrode surface to the deeper double molecular layer [1,2]. Its behavior is complex and relates to the electrode macroscopic and microscopic geometric characteristics [1,3], to the electrolyte proper, and to the operating conditions too, as for example, the applied current intensity or frequency [4,5]. However, in spite of its complexity and of the yet not fully known interface events, this region contains and can supply useful electrochemical information [6-8]. For example, in cellular suspensions, it acts as a highly sensitive transducer to monitor microorganism growth [7-10].

In particular, in Impedance Microbiology [10-12], when interface reactance is being recorded, growth curves typically show a maximum drift at the initial point and thereabouts. Thereafter, the drift slowly decays. Growth curves obtained from the medium bulk, instead, are essentially flat from the very beginning [11]. In the latter reference, such drift effect is barely mentioned. Capacitive growth curves, for example, are used to assess the Minimum Inhibitory Concentration (MIC) of a disinfectant, where either appearance time or decrease to the 20% level is measured [13,14]. Reactance curves, instead of conductance, are more desirable because they show better sensitivity [9]. In milk, several authors have used culture conductance during bacterial growth for quantitative and qualitative assessment of microbial content. Interface capacitance curves C_i _have not been used even though their performance is better by far. The above-mentioned drift has no relationship whatsoever with bacterial growth, it does not stabilize with time, it lacks good reproducibility and introduces a distortion in the temporal curves [6].

In this paper, we show that the drift is due to spurious dc input bias offset currents (I_os_) of the integrated circuits or discrete transistors directly connected to the electrodes in the cells. Such unwanted currents slowly charge up the interface capacitance C_i_, a known phenomenon in the measurement of interface voltages, as for example in pH-meters [15]. Nonetheless, this slow charging current (which can be considered as a spurious dc within the measurement interval) increases the C_i _value [16] and, thus, alters the previous value. In the case of pH, the problem is solved by means of electrometer amplifiers with sub-picoamper I_os_. Commercial equipment to measure impedance does not take care of this problem and the reactance curves often show significant distortions.

To bring down the drift to a negligible level, we have applied three very simple different techniques, i.e., (a) using ultra-low bias current operational amplifiers, (b) using isolating relays instead of analog multiplexers to select the cells, and (c) shortening the time of connection to a minimum and keeping the relays opened after each sample. Our results show that, connecting the circuit to the measurement cell 4 s every 5 min, produced a drift of only 0.17%/hr, turning into negligible the distortion of the growth curves. No temperature effect is seen if its regulation is kept within ± 0.2°C or better.

## Methods

**Figure 1:**
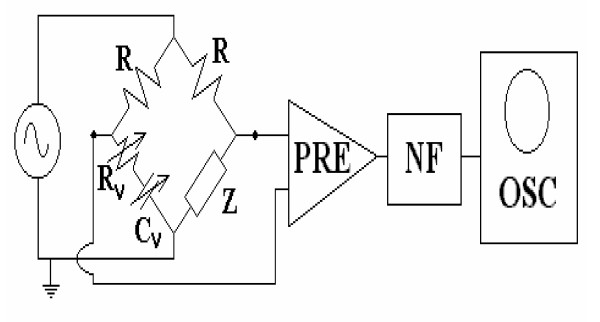
Constant current bridge circuit with replaceable pre-amplifier. Z: measured cell; R: series bridge resistance; R_v_: variable resistor, C_v_: variable capacitor, PRE: preamplifier, NF: 50 Hz notch filter, OSC: oscilloscope.

To quantify the interface reactance (Xi = 1/ ωCi) drift, we used a previously described constant current bridge circuit [6] implemented in such a way that the input preamplifier could be easily replaced (Figure 1).

The cell for the assays with this bridge was a cylindrical glass tube (8 ml volume, 15 mm diameter, 50 mm length), implemented with stainless steel DENTAURUM^® ^wire electrodes (1 mm diameter, 10 mm length) immersed in 0.9% NaCl solution at 37°C. All the measurements were carried out at 120 Hz with an electrode current density of 30 μA/cm^2^. Three different integrated circuits were used as preamplifier testing units: uA741(I_os _= 20 nA, Z_input _= 6 Mohms); LF356(I_os _= 3 pA, Z_input _= 1 Tohms); and AD523(I_os _= 0.25 pA, Z_input _= 10 Tohms). Each was tried with three cells other than the one described above.

Besides, to analyze the effect of the offset current with different instrument systems, culture sterile media and electrodes, we measured X_i _at 120 Hz during 200 min using the following setups,

1) HP4192A, NB broth at (37 ± 0.3)°C, with DENTARUM^® ^wire;

2) HP4284, BHI broth at (37 ± 0.3)°C, with cell C1;

3) FRA-PAR, 0.9% NaCl at 26 ± 1)°C, with cell C2;

4) BACTOMETER, 0.9% NaCl at (37 ± 0.1)°C, with BACTOMETER cell;

where HP4192A stands for a Hewlett-Packard impedance analyzer, HP4284A is a Hewlett-Packard precision LCR meter, FRA-PAR is a system including a 1255 SOLARTRON frequency response analyzer and a 273A PRINCETON APPLIED RESEARCH (PAR) electrochemical interface; BACTOMETER is a patented laboratory custom made impedance microbiology analyzer.

All these four sets measure impedance in the bipolar form. None is internally implemented with input amplifiers in the sub-picoamp range. Current density was ≤ 64 μA/cm^2^. NB and BHI stand, respectively, for Nutritive Broth and Brain Heart Infusion. The culture cells were stabilized for two hours before they were connected to the system.

The cell for the first setup (including DENTAURUM^® ^steel electrodes) was described in a previous paper [10]. The cell named C1 is an acrylic cylindrical cell (100 mm in diameter and 10 mm in length) with two stainless steel AISI 304 electrodes (diameter = 10 mm) polished to 0.05 μm. Cell C2 had the same electrodes but polished to 0.3 μm.

The reasons for choosing 120 Hz can be summarized as follows,

• Low frequency is required to separate R_m _from Z_i _[8].

• At that frequency, and also at 1,000 Hz, X_i _reflects the double layer [17], while higher frequencies do not allow discrimination between R_m _and Z_i_, as stated above.

• From the digital viewpoint, lower frequencies make sampling and further processing on-line easier. Higher frequencies imply conversion frequencies unnecessarily elevated.

As well known, the interface impedance can be characterized in its simplest form by a series equivalent circuit Z_i _= R_i _- j X_i_; herein, we used the series reactance because it carries the same information as the resistive component does, but it is easier to measure [7,10].

To make the I_os _effect negligible, a series of measurements were made with the HP4284A (second setup) by actually disconnecting it after each sample was taken. During the first hour, measurements were made every 5 min, in the second hour every 10 min and, thereafter, every 2 hours. Finally, to monitor the behavior of X_i _as a function of frequency, we used the cylindrical glass tube with DENTAURUM electrodes immersed in saline solution at room temperature with an LF356 as preamplifier and the constant current bridge circuit.

## Results

**Figure 2:**
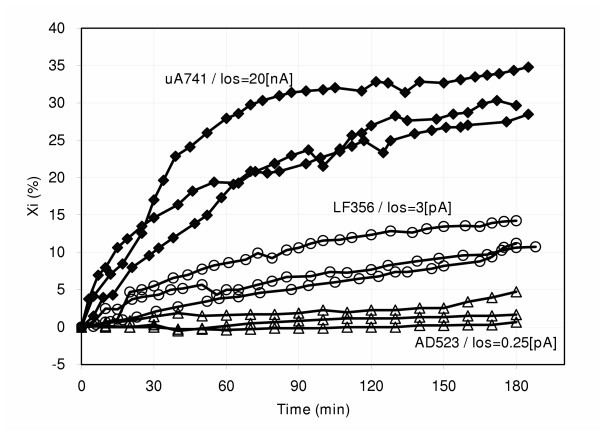
Curves of percent interface reactance versus time using integrated circuits with different offset currents as pre-amplifiers: μA741 (black squares); LF356 (open circles); AD523 (open triangles), from top to bottom. Three curves for each amplifier. There is a large difference in one set of data (for μA741) compared to the other two data sets for the same device. Such difference becomes smaller for LF356 and AD523 as the input bias offset currents further decrease in these devices. This is very likely due to the large industrial variability of these op-amps.

**Figure 3:**
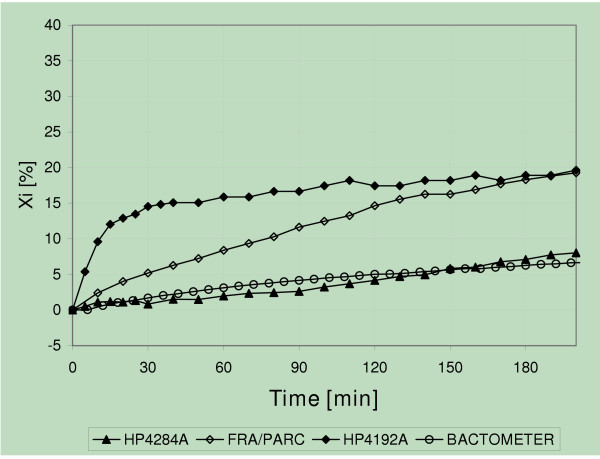
Percent interface reactance versus time using commercial systems. From top to bottom: ◆ Hewlett Packard Impedance Analyzer HP4192A; ◇ FRA or Frequency Response Analyzer 1255, by SOLARTRON, with an electrochemical interface PRINCETON APPLIED RESEARCH (PAR); o BACTOMETER, a patented custom made prototype; ▲ Hewlett Packard Precision LCR meter HP4284A.

The percentage drift curves obtained with each integrated circuit are shown in Figure 2, where it is seen that, as the bias current was increased (upward shift), the interface reactive component appeared with a larger change. The drifts at 60 minutes in Figure 2 are, 28%, 9% and 2%, respectively, for the operational amplifiers μA741, LF356 and AD523. In all cases, the amplifiers with lower bias current produced a lower drift. Figure 3 displays the effects produced when four different equipment were used. At 60 minutes, the drifts came out to be 16% (HP4192A), 8% (FRA/PARC), 4% (BACTOMETER) and 2% (HP4284A). Those magnitudes distort growth curves, especially when either the reactive component or directly the capacitance is plotted.

In Figure 2, there is a large difference in one set of data (for μA741) compared to the other two data sets for the same device. Such difference becomes smaller for LF356 and AD523 as the input bias offset currents further decrease in these devices. This is very likely due to the large industrial variability of these op-amps, which may reach 1000% in the first two types and up to 50% in the last one.

In their book, Firstenberg and Eden [11] show during the initial transient phase an approximated drift of 1% at 60 minutes in a capacitance growth curve of *Escherichia coli *growing in BHI broth. Previous experiments in our laboratory were also marred by a similar effect of 4% at 60 minutes [10].

One possible way of solving the drift problem is to disconnect the culture cell (its electrodes) from the measurement circuit by using a switch relay, during the period when no sample data are collected (dead period). In such situation, the interface capacitance does not charge up. Besides, the longer the interval between measurements, the lower the observed drift. This proposal was evaluated recording the curve of percent interface reactance (X_i_) versus time using the HP4284A and disconnecting the equipment from the cells between measurements. Each sample meant a transient connection to the circuit shorter than 4 s. After 6 hrs, the measured total drift was still in the order of 1%, i.e., essentially negligible.

Another possible solution is the use of a pre-amplifier input circuit of very low bias current (in the sub-picoamp range) which, combined with the in-between OFF periods described above, would reduce the drift even further down.

**Figure 4:**
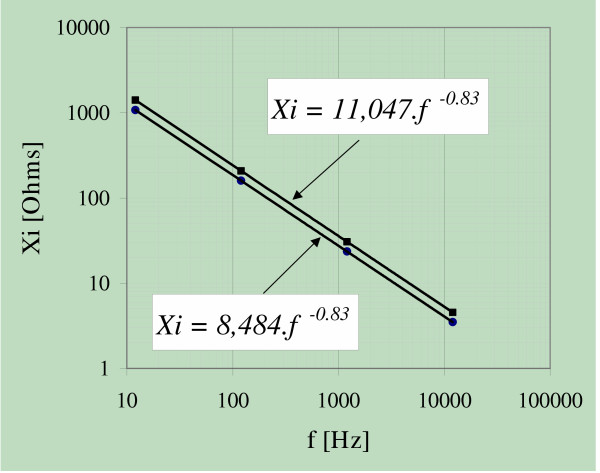
Interface reactance X_i _versus frequency with time as parameter. measurements made at t = 0; + measurements made 3 hours later. The slope did not show any change.

In Figure 4, we studied whether the X_i _changes due to the bias current drift were dependent on the applied frequency. The diagram was plotted at two different times, i.e., at t = 0 and at t = 3 hrs. Regression analysis modeled after a power function led to,

Xi _t = 0 _= A f^B ^= 8,484 f ^-0.83 ^(SD = ± 0.5)    [1]

Xi _t = 3 _= A f^B ^= 11,047 f^-0.83 ^(SD = ± 0.5)    [2]

where SD stands for Standard Deviation. The percent change of X_i _between t = 0 and t = 3 hs was +31% within the analyzed frequency range. This behaviour indicates that the temporal C_i _changes were frequency independent.

## Discussion

The slow drift due to the dc spurious bias current of the input electronic circuitry causes the distortion observed in bacterial growth curves, either of the reactive or resistive type, when the sampling system is connected to the electrodes.

**Figure 5:**
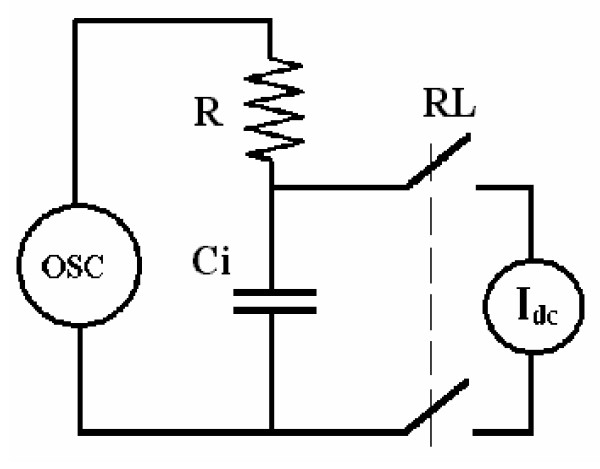
Simplified circuit model of the measuring cell and input preamplifier. R: series resistance; C_i_: interface capacitance; RL: relay contact; I_dc_: unwanted distorting current.

Stainless steel electrodes are considered as polarizable, meaning that they tend to behave as a pure capacitance. Moreover, there are operational amplifiers that behave as constant current sources when connected to a circuit. Such current is the net difference of the input bias currents, which is usually named offset current I_os _[16]; from this application point of view, this is to be considered a spurious unwanted current. The highly simplified model of Figure 5 illustrates the interaction between the circuit electronics and the bipolar measuring cell. Only C_i _is included, because the parallel charge transference resistance is much higher than X_i _[7] at the working frequency. The series resistance R and the oscillator represent the circuitry applying the signal to the cell.

Connecting the preamplifier to the cell (Fig. 5) is equivalent to connecting a constant current dc generator because the input impedance of the amplifier is very high (see Methods Section). Furthermore, since C_i _depends on the interface dc current [18], the larger the absolute value of the current, the higher the value of C_i_. After the circuit is connected, the current through C_i _takes at first a maximum value to decrease exponentially thereafter towards its previous null level. The non-constant capacitance C_i _behaves in a similar way, that is, it is maximal at the beginning and, thereafter, it falls off slowly. Experimentally, this fact is easily seen when stainless steel electrodes are immersed in saline solution or in a culture broth to measure the interface capacitance. Thus, the latter discussion would explain the interface reactance behavior observed in Figure 2; since X_i _is inversely proportional to C_i_, it increases when the current goes down. Frequency is not included here because its influence on X_i _was constant with time.

An important fact is to be underlined. Usually, the first measurement takes place a few seconds after the cell is switched on to the circuitry [7]. Hence, *the initial measured X_i _is not the true interface value*. The true value is the one that existed *before *connection. The deviation of the first erroneous measurement depends on the magnitudes of the spurious continuous current applied by the associated electronics and *the maximum error occurs at the initial moment, when current is maximal*. Hence, precautions should be similar to those taken into account in pH measurements, where electrometer amplifiers are used.

**Figure 6:**
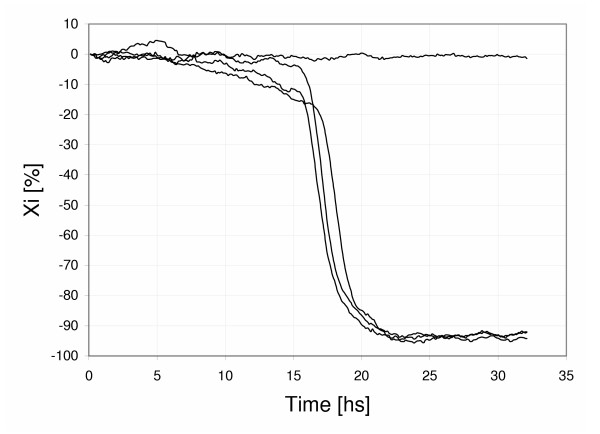
Percent interface reactance X_i _versus time for sulfate reducing bacteria samples growing in Postgate Broth at 37°C. No curve shows any drift, including the reference (sterile) cell.

We believe the I_os _effect is so notorious in Impedance Microbiology because the times during which the cells and the electronic circuitry are connected are too long. A 12 hr growth curve, with a 2%/hr drift, is significantly distorted by the unwanted I_os_; thus, the latter cannot be ignored. In other electrochemical applications such a drift does not show up because the total measurement periods are considerably shorter [19]. In other words, it seems justifiable to use electrometer amplifiers and to decrease connection times between cells and circuitry in order to minimize the drift due to I_os_. The shorter this time, the smaller the continuous current change traversing the interface and the smaller the interface capacitance variation. Results obtained (Fig. 6) with equipment designed having these concepts in mind showed essentially no drift and no distortion in the growth curves [8,9,20,21].

It must be underlined that all the analysis developed herein is only valid for bipolar impedance measurements. We did not make measurements using tripolar or tetrapolar electrode configurations. For this kind of application, the bipolar technique is more practical.

One of the reviewers of this paper perceptively commented that an improved model, which includes a parallel faradic resistance (high in the low frequency range, decreasing with an increase in current density), as well as the half-cell potential, might give a better theoretical background of the study. It brings up an interesting point. The interface is, no doubt and as mentioned above, a complex system produced by the interplay between electrochemical processes and the geometry of the electrode surface. A perfectly smooth surface can be characterized by the double layer capacitance C_dl _in parallel with the series combination of the charge transference resistance R_tc _and the diffusion Warburg impedance Z_w_. The whole set, in turn, in series with the medium resistance R_m _[22,23]. The model becomes rather complex when the surface geometry and the current density are considered [24] and we think that entering into his kind of intricacies exceeds the purpose and intended reach of the article.

Another aspect brought up during the refereeing process referred to the effects of the recording site area or the metal material, even suggesting platinum black as an alternative other than stainless steel. The fact is that the latter does not show any toxic effect, it leads to measurable impedance values for the used geometry and available equipment, and is economic and easy to get without imposing special care. When massive use is the case (as in industrial applications), price becomes of concern, too, and impedance microbiology has a clear industrial facet.

Finally, a few comments should be added regarding the connection time, as another arbiter asked whether measuring for only 4s is really practical for most applications or not. In neural recording, for example, this is certainly not enough time. Our solution has meaning just in impedance microbiology, where bandwidths and sampling frequencies are very low. Let us illustrate with some numbers: Nerve action potentials, with a frequency range of 100 to 2,000 Hz, require a sampling frequency in the order of 8 kHz. Conversely, microbiological growth curves, with signals between 0.00027 Hz and 0.0000016 Hz (a growth curve may take 1 hr up to 7 days to reach its maximum, depending on the kind of bacteria and its culture broth) require sampling frequencies of about 0.0016 Hz (1 sample every 10 minutes). Drift current curves (the main concern of this paper) have similar numerical requirements (signal frequency range, 0.00027 Hz to 0.000027 Hz, which covers drifts that take 1 up to 10 hrs to reach its maximum, and sampling frequency of 0.0016 Hz). Summarizing this point: In impedance microbiology, the signal (that is, the growth curves) and the noise (the drift) coincide in their bandwidth. The method herein described permits a significant noise reduction originated in drift.

## Conclusion

Systems not provided with especially designed input amplifiers introduce drifts leading to unacceptable distortions in bacterial growth curves. Those drifts do depend neither on the measurement conditions nor on the applied frequency. They are due to unwanted input amplifier bias currents connected to the cells. The drifts can be minimized by means of electrometer amplifiers (with sub-picoamps biases) and isolating relays to switch off the cells between measurements.

## Authors' contributions

This paper is the result of over 20 years of experience working in Impedance Microbiology, as a team, always trying to improve the records' quality. Contributions are well balanced and ideas came up slowly after many trials and errors.
